# Supplementary material for: Development of a computer-aided design software for the quantitative evaluation of aesthetic damage
Source: PLoS One. 2019 Dec 18;14(12):e0226322. doi: 10.1371/journal.pone.0226322 (PMC6919621; doi:10.1371/journal.pone.0226322)
Supplement: S1 Code site — 1. (ZIP) [file pone.0226322.s001.zip › [EESC_jr][Projeto]Relatorio_07_21/reports/templates_zip/gentelella-master.zip!/gentelella-master/vendors/DateJS/reports/lcov-report/core/i18n.js.html]

Code coverage report for core/i18n.js


# Code coverage report for core/i18n.js

## Statements: 95.45% (147 / 154)      Branches: 75% (69 / 92)      Functions: 94.12% (32 / 34)      Lines: 95.45% (147 / 154)      Ignored: none

All files » core/ » i18n.js

```
|  |  |  |
| --- | --- | --- |
| 1 2 3 4 5 6 7 8 9 10 11 12 13 14 15 16 17 18 19 20 21 22 23 24 25 26 27 28 29 30 31 32 33 34 35 36 37 38 39 40 41 42 43 44 45 46 47 48 49 50 51 52 53 54 55 56 57 58 59 60 61 62 63 64 65 66 67 68 69 70 71 72 73 74 75 76 77 78 79 80 81 82 83 84 85 86 87 88 89 90 91 92 93 94 95 96 97 98 99 100 101 102 103 104 105 106 107 108 109 110 111 112 113 114 115 116 117 118 119 120 121 122 123 124 125 126 127 128 129 130 131 132 133 134 135 136 137 138 139 140 141 142 143 144 145 146 147 148 149 150 151 152 153 154 155 156 157 158 159 160 161 162 163 164 165 166 167 168 169 170 171 172 173 174 175 176 177 178 179 180 181 182 183 184 185 186 187 188 189 190 191 192 193 194 195 196 197 198 199 200 201 202 203 204 205 206 207 208 209 210 211 212 213 214 215 216 217 218 219 220 221 222 223 224 225 226 227 228 229 230 231 232 233 234 235 236 237 238 239 240 241 242 243 244 245 246 247 248 249 250 251 252 253 254 255 256 257 258 259 260 261 262 263 264 265 266 267 268 269 270 271 272 273 274 275 276 277 278 279 280 281 282 283 284 285 286 287 288 289 290 291 292 293 294 295 296 297 298 299 300 301 302 303 304 305 306 307 308 309 310 311 312 313 314 315 316 317 318 319 320 321 322 323 324 325 326 327 328 329 330 331 332 333 334 335 336 337 338 339 340 341 342 343 344 345 346 347 348 349 350 351 352 353 354 355 356 357 358 359 360 361 362 363 364 365 366 367 368 369 370 371 372 373 374 375 376 377 378 379 380 381 382 383 384 385 386 387 388 389 390 391 392 393 394 395 396 397 398 399 400 401 402 403 404 405 406 407 408 409 410 411 412 413 414 415 416 | 1 1 1 1 1   28001 28001 26062   1939   28001 7360   28001     480 480 10240 10240     480     320 320 9120 9120     320     960 960 8320 8320     960     1939 1939   3 3   3 3   3 3   3 3   160 160   1767 1767 1767 1767   214 214 214     1767   1939     7360 7360 6123   1237   7360       1 160 1600 1600         1 1601 1601 1601 1280 960   320     321       1   1 1 1 1   1 1       1 1 1 1       1 1     1   1 1 1             1   160 160 1600 1600     160     160                                       160     160                                                                                 160     160 160 160 6240 6240     160 2880 2880     160     160     160     160     160     160     160     160                           160                                                                                                     1 160                     160                         160 160 160     1   321     637     159 159 157 157 157 157   2 2                     2   1 1 1 1 1 1 1 1 1   1 1       1 1       158 158 158   158 156             2     1 | ``` (function () { 	var $D = Date; 	var lang = Date.CultureStrings ? Date.CultureStrings.lang : null; 	var loggedKeys = {}; // for debug purposes. 	var getText = { 		getFromKey: function (key, countryCode) { 			var output; 			if (Date.CultureStrings && Date.CultureStrings[countryCode] && Date.CultureStrings[countryCode][key]) { 				output = Date.CultureStrings[countryCode][key]; 			} else { 				output = getText.buildFromDefault(key); 			} 			if (key.charAt(0) === "/") { // Assume it's a regex 				output = getText.buildFromRegex(key, countryCode); 			} 			return output; 		}, 		getFromObjectValues: function (obj, countryCode) { 			var key, output = {}; 			for(key in obj) { 				Eif (obj.hasOwnProperty(key)) { 					output[key] = getText.getFromKey(obj[key], countryCode); 				} 			} 			return output; 		}, 		getFromObjectKeys: function (obj, countryCode) { 			var key, output = {}; 			for(key in obj) { 				Eif (obj.hasOwnProperty(key)) { 					output[getText.getFromKey(key, countryCode)] = obj[key]; 				} 			} 			return output; 		}, 		getFromArray: function (arr, countryCode) { 			var output = []; 			for (var i=0; i < arr.length; i++){ 				Eif (i in arr) { 					output[i] = getText.getFromKey(arr[i], countryCode); 				} 			} 			return output; 		}, 		buildFromDefault: function (key) { 			var output, length, split, last; 			switch(key) { 				case "name": 					output = "en-US"; 					break; 				case "englishName": 					output = "English (United States)"; 					break; 				case "nativeName": 					output = "English (United States)"; 					break; 				case "twoDigitYearMax": 					output = 2049; 					break; 				case "firstDayOfWeek": 					output = 0; 					break; 				default: 					output = key; 					split = key.split("_"); 					length = split.length; 					if (length > 1 && key.charAt(0) !== "/") { 						// if the key isn't a regex and it has a split. 						last = split[(length - 1)].toLowerCase(); 						Eif (last === "initial" \|\| last === "abbr") { 							output = split[0]; 						} 					} 					break; 			} 			return output; 		}, 		buildFromRegex: function (key, countryCode) { 			var output; 			if (Date.CultureStrings && Date.CultureStrings[countryCode] && Date.CultureStrings[countryCode][key]) { 				output = new RegExp(Date.CultureStrings[countryCode][key], "i"); 			} else { 				output = new RegExp(key.replace(new RegExp("/", "g"),""), "i"); 			} 			return output; 		} 	};   	var shallowMerge = function (obj1, obj2) { 		for (var attrname in obj2) { 			Eif (obj2.hasOwnProperty(attrname)) { 				obj1[attrname] = obj2[attrname]; 			} 		} 	};   	var __ = function (key, language) { 		var countryCode = (language) ? language : lang; 		loggedKeys[key] = key; 		if (typeof key === "object") { 			if (key instanceof Array) { 				return getText.getFromArray(key, countryCode); 			} else { 				return getText.getFromObjectKeys(key, countryCode); 			} 		} else { 			return getText.getFromKey(key, countryCode); 		} 	}; 	 	var loadI18nScript = function (code) { 		// paatterned after jQuery's getScript. 		var url = Date.Config.i18n + code + ".js"; 		var head = document.getElementsByTagName("head")[0] \|\| document.documentElement; 		var script = document.createElement("script"); 		script.src = url;   		var completed = false; 		var events = { 			done: function (){} // placeholder function 		}; 		// Attach handlers for all browsers 		script.onload = script.onreadystatechange = function() { 			Eif ( !completed && (!this.readyState \|\| this.readyState === "loaded" \|\| this.readyState === "complete") ) { 				events.done(); 				head.removeChild(script); 			} 		};   		setTimeout(function() { 			head.insertBefore(script, head.firstChild); 		}, 0); // allows return to execute first 		 		return { 			done: function (cb) { 				events.done = function() { 					Eif (cb) { 						setTimeout(cb,0); 					} 				}; 			} 		}; 	};   	var buildInfo = { 		buildFromMethodHash: function (obj) { 			var key; 			for(key in obj) { 				Eif (obj.hasOwnProperty(key)) { 					obj[key] = buildInfo[obj[key]](); 				} 			} 			return obj; 		}, 		timeZoneDST: function () { 			var DST = { 				"CHADT": "+1345", 				"NZDT": "+1300", 				"AEDT": "+1100", 				"ACDT": "+1030", 				"AZST": "+0500", 				"IRDT": "+0430", 				"EEST": "+0300", 				"CEST": "+0200", 				"BST": "+0100", 				"PMDT": "-0200", 				"ADT": "-0300", 				"NDT": "-0230", 				"EDT": "-0400", 				"CDT": "-0500", 				"MDT": "-0600", 				"PDT": "-0700", 				"AKDT": "-0800", 				"HADT": "-0900" 			}; 			return __(DST); 		}, 		timeZoneStandard: function () { 			var standard = { 				"LINT": "+1400", 				"TOT": "+1300", 				"CHAST": "+1245", 				"NZST": "+1200", 				"NFT": "+1130", 				"SBT": "+1100", 				"AEST": "+1000", 				"ACST": "+0930", 				"JST": "+0900", 				"CWST": "+0845", 				"CT": "+0800", 				"ICT": "+0700", 				"MMT": "+0630", 				"BST": "+0600", 				"NPT": "+0545", 				"IST": "+0530", 				"PKT": "+0500", 				"AFT": "+0430", 				"MSK": "+0400", 				"IRST": "+0330", 				"FET": "+0300", 				"EET": "+0200", 				"CET": "+0100", 				"GMT": "+0000", 				"UTC": "+0000", 				"CVT": "-0100", 				"GST": "-0200", 				"BRT": "-0300", 				"NST": "-0330", 				"AST": "-0400", 				"EST": "-0500", 				"CST": "-0600", 				"MST": "-0700", 				"PST": "-0800", 				"AKST": "-0900", 				"MIT": "-0930", 				"HST": "-1000", 				"SST": "-1100", 				"BIT": "-1200" 			}; 			return __(standard); 		}, 		timeZones: function (data) { 			var zone; 			data.timezones = []; 			for (zone in data.abbreviatedTimeZoneStandard) { 				Eif (data.abbreviatedTimeZoneStandard.hasOwnProperty(zone)) { 					data.timezones.push({ name: zone, offset: data.abbreviatedTimeZoneStandard[zone]}); 				} 			} 			for (zone in data.abbreviatedTimeZoneDST) { 				Eif (data.abbreviatedTimeZoneDST.hasOwnProperty(zone)) { 					data.timezones.push({ name: zone, offset: data.abbreviatedTimeZoneDST[zone], dst: true}); 				} 			} 			return data.timezones; 		}, 		days: function () { 			return __(["Sunday", "Monday", "Tuesday", "Wednesday", "Thursday", "Friday", "Saturday"]); 		}, 		dayAbbr: function () { 			return __(["Sun", "Mon", "Tue", "Wed", "Thu", "Fri", "Sat"]); 		}, 		dayShortNames: function () { 			return __(["Su", "Mo", "Tu", "We", "Th", "Fr", "Sa"]); 		}, 		dayFirstLetters: function () { 			return __(["S_Sun_Initial", "M_Mon_Initial", "T_Tues_Initial", "W_Wed_Initial", "T_Thu_Initial", "F_Fri_Initial", "S_Sat_Initial"]); 		}, 		months: function () { 			return __(["January", "February", "March", "April", "May", "June", "July", "August", "September", "October", "November", "December"]); 		}, 		monthAbbr: function () { 			return __(["Jan_Abbr", "Feb_Abbr", "Mar_Abbr", "Apr_Abbr", "May_Abbr", "Jun_Abbr", "Jul_Abbr", "Aug_Abbr", "Sep_Abbr", "Oct_Abbr", "Nov_Abbr", "Dec_Abbr"]); 		}, 		formatPatterns: function () { 			return getText.getFromObjectValues({ 				shortDate: "M/d/yyyy", 				longDate: "dddd, MMMM dd, yyyy", 				shortTime: "h:mm tt", 				longTime: "h:mm:ss tt", 				fullDateTime: "dddd, MMMM dd, yyyy h:mm:ss tt", 				sortableDateTime: "yyyy-MM-ddTHH:mm:ss", 				universalSortableDateTime: "yyyy-MM-dd HH:mm:ssZ", 				rfc1123: "ddd, dd MMM yyyy HH:mm:ss", 				monthDay: "MMMM dd", 				yearMonth: "MMMM, yyyy" 			}, Date.i18n.currentLanguage()); 		}, 		regex: function () { 			return getText.getFromObjectValues({ 				inTheMorning: "/( in the )(morn(ing)?)\\b/", 				thisMorning: "/(this )(morn(ing)?)\\b/", 				amThisMorning: "/(\b\\d(am)? )(this )(morn(ing)?)/", 				inTheEvening: "/( in the )(even(ing)?)\\b/", 				thisEvening: "/(this )(even(ing)?)\\b/", 				pmThisEvening: "/(\b\\d(pm)? )(this )(even(ing)?)/", 				jan: "/jan(uary)?/", 				feb: "/feb(ruary)?/", 				mar: "/mar(ch)?/", 				apr: "/apr(il)?/", 				may: "/may/", 				jun: "/jun(e)?/", 				jul: "/jul(y)?/", 				aug: "/aug(ust)?/", 				sep: "/sep(t(ember)?)?/", 				oct: "/oct(ober)?/", 				nov: "/nov(ember)?/", 				dec: "/dec(ember)?/", 				sun: "/^su(n(day)?)?/", 				mon: "/^mo(n(day)?)?/", 				tue: "/^tu(e(s(day)?)?)?/", 				wed: "/^we(d(nesday)?)?/", 				thu: "/^th(u(r(s(day)?)?)?)?/", 				fri: "/fr(i(day)?)?/", 				sat: "/^sa(t(urday)?)?/", 				future: "/^next/", 				past: "/^last\|past\|prev(ious)?/", 				add: "/^(\\+\|aft(er)?\|from\|hence)/", 				subtract: "/^(\\-\|bef(ore)?\|ago)/", 				yesterday: "/^yes(terday)?/", 				today: "/^t(od(ay)?)?/", 				tomorrow: "/^tom(orrow)?/", 				now: "/^n(ow)?/", 				millisecond: "/^ms\|milli(second)?s?/", 				second: "/^sec(ond)?s?/", 				minute: "/^mn\|min(ute)?s?/", 				hour: "/^h(our)?s?/", 				week: "/^w(eek)?s?/", 				month: "/^m(onth)?s?/", 				day: "/^d(ay)?s?/", 				year: "/^y(ear)?s?/", 				shortMeridian: "/^(a\|p)/", 				longMeridian: "/^(a\\.?m?\\.?\|p\\.?m?\\.?)/", 				timezone: "/^((e(s\|d)t\|c(s\|d)t\|m(s\|d)t\|p(s\|d)t)\|((gmt)?\\s*(\\+\|\\-)\\s*\\d\\d\\d\\d?)\|gmt\|utc)/", 				ordinalSuffix: "/^\\s*(st\|nd\|rd\|th)/", 				timeContext: "/^\\s*(\\:\|a(?!u\|p)\|p)/" 			}, Date.i18n.currentLanguage()); 		} 	};   	var CultureInfo = function () { 		var info = getText.getFromObjectValues({ 			name: "name", 			englishName: "englishName", 			nativeName: "nativeName", 			amDesignator: "AM", 			pmDesignator: "PM", 			firstDayOfWeek: "firstDayOfWeek", 			twoDigitYearMax: "twoDigitYearMax", 			dateElementOrder: "mdy" 		}, Date.i18n.currentLanguage());   		var constructedInfo = buildInfo.buildFromMethodHash({ 			dayNames: "days", 			abbreviatedDayNames: "dayAbbr", 			shortestDayNames: "dayShortNames", 			firstLetterDayNames: "dayFirstLetters", 			monthNames: "months", 			abbreviatedMonthNames: "monthAbbr", 			formatPatterns: "formatPatterns", 			regexPatterns: "regex", 			abbreviatedTimeZoneDST: "timeZoneDST", 			abbreviatedTimeZoneStandard: "timeZoneStandard" 		});   		shallowMerge(info, constructedInfo); 		buildInfo.timeZones(info); 		return info; 	};   	$D.i18n = { 		__: function (key, lang) { 			return __(key, lang); 		}, 		currentLanguage: function () { 			return lang \|\| "en-US"; 		}, 		setLanguage: function (code, force, cb) { 			var async = false; 			if (force \|\| code === "en-US" \|\| (!!Date.CultureStrings && !!Date.CultureStrings[code])) { 				lang = code; 				Date.CultureStrings = Date.CultureStrings \|\| {}; 				Date.CultureStrings.lang = code; 				Date.CultureInfo = new CultureInfo(); 			} else { 				Eif (!(!!Date.CultureStrings && !!Date.CultureStrings[code])) { 					Iif (typeof exports !== "undefined" && this.exports !== exports) { 						// we're in a Node enviroment, load it using require 						try { 							require("../i18n/" + code + ".js"); 							lang = code; 							Date.CultureStrings.lang = code; 							Date.CultureInfo = new CultureInfo(); 						} catch (e) { 							// var str = "The language for '" + code + "' could not be loaded by Node. It likely does not exist."; 							throw new Error("The DateJS IETF language tag '" + code + "' could not be loaded by Node. It likely does not exist."); 						} 					} else if (Date.Config && Date.Config.i18n) { 						// we know the location of the files, so lets load them					 						async = true; 						loadI18nScript(code).done(function(){ 							lang = code; 							Date.CultureStrings = Date.CultureStrings \|\| {}; 							Date.CultureStrings.lang = code; 							Date.CultureInfo = new CultureInfo(); 							$D.Parsing.Normalizer.buildReplaceData(); // because this is async 							Eif ($D.Grammar) { 								$D.Grammar.buildGrammarFormats(); // so we can parse those strings... 							} 							Eif (cb) { 								setTimeout(cb,0); 							} 						}); 					} else { 						Date.console.error("The DateJS IETF language tag '" + code + "' is not available and has not been loaded."); 						return false; 					} 				} 			} 			$D.Parsing.Normalizer.buildReplaceData(); // rebuild normalizer strings 			Eif ($D.Grammar) { 				$D.Grammar.buildGrammarFormats(); // so we can parse those strings... 			} 			if (!async && cb) { 				setTimeout(cb,0); 			} 		}, 		getLoggedKeys: function () { 			return loggedKeys; 		}, 		updateCultureInfo: function () { 			Date.CultureInfo = new CultureInfo(); 		} 	}; 	$D.i18n.updateCultureInfo(); // run automatically }()); ``` |
```

Generated by istanbul at Sun Nov 16 2014 12:08:33 GMT-0800 (PST)
